# Effects of the interactions between platelets with other cells in tumor growth and progression

**DOI:** 10.3389/fimmu.2023.1165989

**Published:** 2023-04-17

**Authors:** Yaxin Li, Haiyan Wang, Zhen Zhao, Yuanming Yang, Zifan Meng, Lifeng Qin

**Affiliations:** Department of Blood Transfusion, The Affiliated Hospital of Qingdao University, Qingdao, China

**Keywords:** platelets, immune cells, interaction, tumorigenesis, tumor development

## Abstract

It has been confirmed that platelets play a key role in tumorigenesis. Tumor-activated platelets can recruit blood cells and immune cells to migrate, establish an inflammatory tumor microenvironment at the sites of primary and metastatic tumors. On the other hand, they can also promote the differentiation of mesenchymal cells, which can accelerate the proliferation, genesis and migration of blood vessels. The role of platelets in tumors has been well studied. However, a growing number of studies suggest that interactions between platelets and immune cells (e.g., dendritic cells, natural killer cells, monocytes, and red blood cells) also play an important role in tumorigenesis and tumor development. In this review, we summarize the major cells that are closely associated with platelets and discuss the essential role of the interaction between platelets with these cells in tumorigenesis and tumor development.

## 1 Introduction

Tumorigenesis and tumor development are not only manifested in the malignant proliferation of tumor cells, but also include their immune escape and metastasis. The tumor microenvironment has an extremely important influence on it. The mechanism of platelet action in the tumor microenvironment has been well explored, but the exact underlying mechanisms and implications of platelet interactions with other cells in tumor development and progression remain unknown. We endeavored to summarize the current literature in order to dissect the specific role of platelets in tumors.

Platelets are the main players in primary and secondary hemostasis. It has been discovered that platelets also play an crucial role in tumorigenesis and tumor development. On the one hand, tumor cells induce platelet aggregation, which promotes the release of angiogenic factors and therefore further facilitating cancer progression (1). In contrast, platelets that have been “educated” by cancer cells can promote cancer development by interacting with other cells. Specifically, the platelets that activated by tumor cells can recruit immune cells and blood cells to migrate to the tumor site, modulate the inflammatory environment at the primary and metastatic tumor sites, and promote proliferation and metastasis of tumor cells (2). Platelets also regulate the effector functions of immune cells, promote antigen presentation in dendritic cells, recruit and differentiate monocytes, promote the formation of neutrophil extracellular traps, activates immune responses, and reduce the barrier effect of natural killer (NK) cells (3–6). Moreover, tumor-activated platelets stimulate the division of vascular endothelial cells; enhance the permeability of vascular endothelial cells and promote angiogenesis and migration of tumor cells (7). Finally, platelets can also promote thrombosis by interacting with red blood cells and facilitating the initiation of the clotting cascade, thereby increasing the risk of death in cancer patients (8).

## 2 Platelet-tumor cell interactions in tumor development

Platelets are small fragments of anucleated cells which are released from megakaryocytes (MKs). Beyond their traditional roles in thrombosis and hemostasis, platelets are also identified as key mediators of malignancy. Cancer patients are usually accompanied by excessive thrombocytosis and platelet activation (9–11).Tumor cells activate platelets through different stimuli, while platelets promote tumor cell proliferation, intracellular signaling and EMT through various mediators. Platelet-tumor cell interactions play an important role in tumor growth, thrombosis, angiogenesis, dissemination, metastasis and immune escape (12, 13). Platelets have been shown to exert their impact in multiple ways, and tumors, in turn, can also influence the platelet number, behavior, and even phenotype by a process that is called platelet “education” (14). Platelets have the following main roles in tumor microenvironment:(1) Contributes to the growth of tumors. Platelets have a large number of growth factors in their alpha granules (15–18). They can be present in the tumor microenvironment and contact with tumor cells directly (19, 20). When activated, platelets secrete vascular endothelial growth factor (VEGF) (21, 22), transforming growth factor β (TGF-β)and platelet-derived growth factor (PDGF) (23) promoting induction of tumor growth, angiogenesis and tumor neovascularization (24–26). (2) facilitation of the tumor thrombosis. Armand Trousseau first described in 1865 that tumor cells could induce thrombosis formation (named Trousseau’s syndrome) (27). Tumor cells can promote platelet activation by stimulating platelet aggregation, leads to a phenomenon that is known as tumor cell-induced platelet aggregation (TCIPA) (28). TCIPA is not only associated with a very high risk of thrombosis, but is also thought to promote angiogenesis and metastasis and is thus considered to be inversely associated with prognosis and survival (29, 30). During TCIPA, platelets express many selectins and integrins that attach to Circulating tumor cells (CTCs) to protect tumor cells to achieve immune escape (31–33). (3)Induction of tumor angiogenesis. Platelets have the ability to induce tumor angiogenesis. Platelets can deliver a variety of pro-angiogenic factors to tumors as well as stimulate tumor cells to express pro-angiogenic factors, including PDGF, VEGF, MMP and FGF (34). Platelets are actively recruited by cancer cells *via* P-selectin (35). When platelets arrive at the tumor microenvironment they will adhere to the cancer cell membrane and stimulate VEGF secretion, promote cancer cell proliferation and angiogenesis (36). Not only that, animal models confirm that tumor-educated platelets stimulate angiogenesis more efficient than normal platelets, by delivering pro-angiogenic factors more efficiently (37). (4)Promotes tumor cell invasion and metastasis and escape immune surveillance. An important strategy for cancer cell survival in the circulation is interaction with platelets, and almost all cancer metastatic processes seem to be mediated through tumor cell-platelet interactions (38). In tumor microenvironment, expression of PD-1-PD-L1 ligand by tumor cells and other stromal cells inhibit T cells and helps tumor cells evade immune surveillance. Tumor cells with high PD-L1 expression are able to fight cancer immunosurveillance (39, 40). PD-L1 expression in tumors is associated with poorer prognosis in various cancers (41). It has been suggested that PD-L1 is reported to be highly expressed on platelets from cancer patients and is lacking on normal platelets (42–44), which is thought to be the result of PD-L1 transfered from cancer cells to platelets (39). However, more studies have demonstrated that normal platelets also express PD-L1. Zaslavsky et al. demonstrated the presence of PD-L1 in platelets from both healthy individuals and patients with advanced cancer using Western blot and confirmed the contribution of PD-L1-expressing platelets to overall PD-L1 expression in tumors (45, 46). They demonstrated that platelet attach to PD-L1-negative tumor cells resulted in PD-L1 protein expression and platelets can modulate tumor cells to evade immune surveillance by expressing PD-L1 (47). They also observed that *in vitro*, anti-platelet drugs such as aspirin can neutralize the inhibitory effect of platelets on T cell-mediated cytotoxicity (45). Rolfes et al. also detected PD-L1 on platelets isolated from patients with head and neck squamous cell carcinoma (HNSCC) and confirmed that platelets from healthy donors also express small amount PD-L1. Not only that, they observed significantly higher PD-L1 expression in platelets of cancer-free individuals who smoked regularly (42) and suggested that PD-L1 may predict early lung cancer (48). These studies have confirmed that platelets express PD-L1 and can promote PD-L1 expression in tumors. Platelets increase the expression of PD-L1 in cancer cells mainly through a number of signaling pathways: Platelets and platelet secretion products can activate NF-κB, TGF-β/Smad and JAK/STAT pathways in tumor cells (49), which also increase the expression of PD-L1 in cancer cells exposured to platelets directly through Smad2/3 and NF-κB signaling pathways, and increases PD-L1 mostly through the TGFβR1/Smad signaling pathway exposured to platelets indirectly (45). Platelets also stimulate the release of metalloproteinases from tumor cells, and in turn contributes to tumor cell invasion by accelerating extracellular matrix degradation (50, 51). In addition, activated platelets secrete lysophosphatidic acid, which upregulates matrix metalloproteinase 2 (MMP2), MMP7, and MMP9 activities in cancer cells, then promote cancer progression by facilitating the detachment of cancer cells from their primary site and into the circulation (52). When tumor cells leave tumor microenvironment and reach the circulation, they are subject to high shear stress of the immune system (53). At this phase, tumor cells express megakaryocyte genes and platelet surface markers to mimic platelets (54, 55), and platelets can transfer major histocompatibility complex (MHC) class I to the tumor cells, giving tumor cells a false “pseudo-normal” appearance and thus evading natural killer cell (56). The capacity of platelets to prevent circulating tumor cells from attacking by the immune system may contribute significantly to the metastatic process (57, 58).

## 3 Platelet- neutrophil interactions in tumors

Tumor development is a complicated process that depends on several pivotal hallmarks, such as the induction of angiogenesis, promotionof inflammation, evasion of immune destruction, and activation of invasion and metastasis. Herein, platelet-leukocyte interactions play a crucial role (59).Platelets play a critical role in promoting thrombosis and inflammation progression, linking hemostasis and immune responses (60, 61). They can also be viewed as an extension of the cellular immune system (3, 62–65)as they play a crucial role in modulating leukocyte function and the release of inflammatory signals.

### 3.1 Platelet- neutrophil interactions promote inflammation in the tumor microenvironment

**Figure 1:**
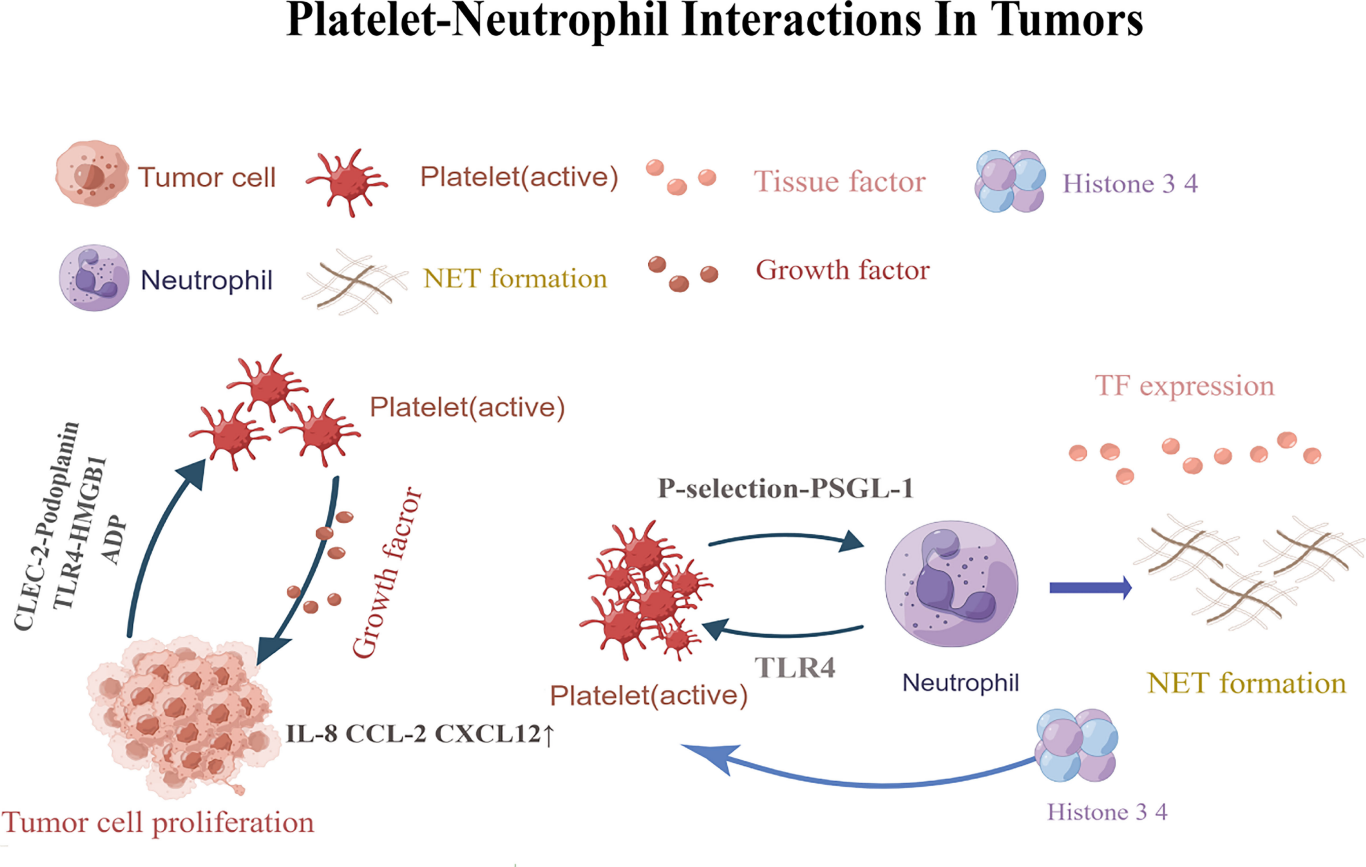
Platelets are activated by CLEC-2-podoplanin and TLR4-HMGB1.Activated platelets release factors that promote adhesion and angiogenesis, and increaseIL-8, CXCL12,and CCL2 levels. Eventually leading to tumor cell angiogenesis and tumor proliferation. P-selectin-PSGL-1 promotes the formation of neutrophil traps. TLR4-induced platelet activation can induce platelets to bind to neutrophils and form robust NETs, releasing histones 3 and 4, which further activate circulating platelets and promote the formation of more NETs.CLEC-2,C-type lectin-like immune receptor 2; TLR4,Toll-like receptor; HMGB1,high-mobility group box 1; PSGL-1,P-selectin glycoprotein ligand 1; NETs, Neutrophil extracellular traps. This figure is drawn by Figdraw (https://figdraw.com/). The "↑" arrows refer to the increased expression of IL-8, CXCL12, and CCL2.

Tumors are not simply clusters of malignant cells, but complex structures composed of malignant,non-transformed cells, and the tumor microenvironment (66–71). It was found that the TEM contains a large number of different types of cells, including fibroblasts, vascular endothelial cells, adipocytes, stromal cells and immune cells such as B lymphocytes, T lymphocytes, tumor-associated macrophages and NK cells. Most cells in the TEM can produce cytokines and play an important role in promoting or suppressing tumors (72). The tumor microenvironment contains a large number of inflammatory cells and pro-inflammatory mediators (such as cytokines, chemokines and prostaglandins) that are capable of participating in a variety of inflammatory responses and acting on malignant cells in an autocrine and/or paracrine manner (73). Platelet- neutrophil interactions act as pro-inflammatory agents in the tumor microenvironment through various mechanisms (**Figure 1**). First, platelet- neutrophil interactions are mediated by P-selectin (or CD62P)-P-selectin glycoprotein ligand 1 (PSGL-1) and further stabilized by platelet glycoprotein (GP) Ib, glycoprotein (GP)IIb/IIIa, intercellular adhesion molecule-2 (ICAM-2), and the CD40 ligand (CD40L). Platelets also interact with leukocytes to form platelet- neutrophil aggregates that circulate in the blood, promoting leukocyte recruitment, activation, extravasation, phenotypic switching, and other functional changes (2, 3). Furthermore, platelets are activated upon injury and directly contact pathogens *via* their Toll-like receptors (TLRs) (2). TLR binding triggers the expression of several adapter proteins and downstream kinases induces the expression of essential pro-inflammatory mediators, activates the innate immune response, and promotes the maturation of adaptive immune components (74). Moreover, activated platelets release granule contents that regulate the tumor microenvironment, such as pro-inflammatory cytokines (30). These platelet-released pro-inflammatory cytokines are powerful activators and recruiters of leukocytes which in turn contribute to the immunomodulatory effects of platelets (75, 76). Examples of these chemokines include chemokine ligand 1 (CXCL1),CXCL4,CXCL5,CXCL7, CXCL12,interleukin-8 (IL-8),and transforming growth factorβ (TGF-β) (30, 77).

### 3.2 Platelet- neutrophil cell interactions promote tumor thrombosis and angiogenesis

The strong association between thrombosis and cancer has been known since 1865 when Armand Trousseau first confirmed that localized cancers could lead to venous thrombosis at distant sites (27, 78). The risk of venous thromboembolism in patients with cancer is four- to seven-fold more than in healthy individuals. Cancer-related thrombosis is a cause of high cancer morbidity and mortality (79). The main reason of thrombosis in cancer patients is t platelets activated by cancer cells (78, 80). The interaction between platelets and leukocytes, such as the activation of tissue factor (TF) and G proteins and the promotion of neutrophil trap formation and platelet activation, plays a crucial role in promoting tumor thrombosis. The hypercoagulable state is caused by multiple factors, including changes in the expression of circulating pro-coagulation proteins and systemic inflammation, including TF, fibrinogen, factor V (FV), FVI, FVIII, and FX (81, 82). First, TF is normally expressed in an inactive form on the cell membrane of tumor cells, and platelets play a key role in activating TF. Direct interaction with TF-expressing leukocytes and platelets can strengthen TF activity and promote coagulation (83). Additionally, tumor cells show increased vascular permeability and upregulated TF expression (84). Once it comes in contact with activator factor VIIa in the bloodstream, TF is arguably the primary activator of the coagulation cascade (15, 85, 86). It is commonly expressed in cancer cells and cancer-derived particles (15, 16, 85–87). The origin and significance of blood-derived tissue factor (TF) is widely debated. Monocytes have long been considered as the main source of blood-derived TF (88–91). Some studies have identified TF proteins in platelets by flow cytometry, ELISA, Western blotting, immunocytochemistry and immunoelectron microscopy (92–94). TF in platelets is present in α-granules and also in the open tubule system (95, 96) and is hopefully output to the plasma membrane upon activation (95). Reports strongly suggest that the initiation of platelet PCA is dependent on platelet activation-induced ab initio synthesis of TF. TF expressed by platelets is probably through three proven mechanisms, namely: (1) FusionTF-containing microparticles (MPs) with activated platelet membranes; (2) stored in platelet α-granules and extravasates to the cell surface after platelet activation;(3) Activated platelets synthesize surface TF from *de novo* after splicing the pre-TF mRNA (97). Human platelets, a new type of TF-synthesizing cell, will act as “TF-bearing cells”, ensuring that TF is localized at the right time and place, thus promoting coagulation with greater rational precision (98).

In addition to the TF, neutrophil extracellular traps (NETs) have also been shown to have a strong relationship with thrombosis. The association between thrombosis and neutrophil extracellular traps(NETs) was first demonstrated several years ago by Fuchs et al (82). Platelets play a huge role in the formation of NETs. Firstly, the pro-thrombotic effect of NETs can be explained by their high histone and nucleic acid content, rendering them highly pro-coagulant with the capacity to activate and aggregate platelets (82). Interaction between platelet P-selectin and neutrophil P-selectin glycoprotein ligand 1 (PSGL-1) promotes neutrophil activation and migration, leading to IL-8 release and the formation of neutrophil extracellular traps (NETs) (5, 99). NETs, in turn, recruit more platelets by presenting histones at platelet TLR4,leading to its activation, andre-initiation of a pro-coagulant environment that promotes thrombosis (81, 100). Besides, one key damage-associated molecular pattern (DAMP) expressed and released by platelets is High Mobility Group Box 1 (HMGB1). HMGB1 is a highly conserved, non-histone chromosomal protein that has pro-inflammatory and pro-coagulant functions and mediates NET formation in venous thrombosis (101–103) and tumor (104). HMGB1 induces NETosis through C-X-C motif chemokine receptor 4 (CXCR4) and Toll-like receptor 4 (TLR4),and can be inhibited by neutralizing HMGB1 antibodies (105, 106). HMGB1 induces NETosis and activates platelets; it is also released from activated platelets and extruded from NETosed neutrophils. Extruded HMGB1 in turn, induces platelet activation, thrombosis and further aggravates NETosis (107). Therefore, HMGB1 acts as the center of the crosstalk between thrombosis and NETosis and mediates the exacerbation cascade caused by this crosstalk. Moreover,HMGB1 signals is thought to exert tumor-promoting effects in malignant tumors *via* the tumor HMGB1/RAGE axis through agonist receptors, such as receptors for advanced glycosylation end products (RAGE), and other pattern recognition receptors, like TLR2, TLR4, and TLR9 (108, 109). It has been demonstrated that tumor growth is accompanied by elevated HMGB which promotes cancer cell survival and protects cells from the cytotoxic effects of adriamycin in homozygous mice (110). Finally, neutrophil- derived extracellular DNA provides scaffolding for platelet activation (82) and is indispensable for the spread of deep-vein thrombosis due to its ability to bind and activate FXII (111). In addition, tumor cells and its derived micro-particles are able to trigger NET formation directly or indirectly by activating platelets, which leads to a further activation of platelet and release of TF (112). Recent clinical evidence suggests a definite link between NET formation and thrombosis in in tumor patients. These data indicate that NETs promote the development of venous thromboembolism (VTE) in these patients with tumor (113).

Platelet-neutrophil interactions promote not only tumor thrombosis, but also tumor angiogenesis. Platelets are locally activated through the interaction of podoplanin with C-type lectin-like immune receptor 2 (CLEC-2),high-mobility group box 1 (HMGB1) with TLR4 (13, 114). Activated platelets release factors that promote cell adhesion and angiogenesis, leading to tumor proliferation. They also release growth factors and upregulate cytokine release from leukocytes, endothelial cells, and tumor cells, which increases the local tissue IL-8,CXCL12andCCL2levels, promoting cell proliferation and tumor angiogenesis (86).

### 3.3 Platelet- neutrophil interactions promote tumor metastasis

In addition to their role in thrombosis, NETs are also involved in tumor metastasis. Research has shown that the deposition of neutrophil traps *in vivo* increased the adhesion of tumor cells to the liver and lung microvasculature, leading to tumor cell migration and invasion (87). In contrast, it was found that NETs formed in the absence of systemic inflammation increased tumor adhesion *in vivo (* 82, 100). Finally, it has also been documented that NETs are responsible for awakening dormant cancers, promoting a pro-cancer microenvironment that leads to tumor metastasis (115). Not only that, the interaction between platelets and neutrophils enhances their cytotoxicity by increasing reactive oxygen species production (116). At later stages of tumor development, neutrophils promote tumor cell metastasis by inhibiting NK cells (78) and cytotoxic T-cells (83).

## 4 Platelet-erythrocyte interactions in tumors

Erythrocytes, or red blood cells (RBCs), have a major influence on blood coagulation, hemostasis, and thrombosis and have been a major focus of hematology, hemostasis, and thrombosis research (117). The role of erythrocyte-platelet interactions in tumors has also received increasing attention. Anemia is an independent poor prognostic factor for patients with various malignancies, adversely affecting their survival and quality of life (118). Studies have shown that tumor embolization, vascular endothelial damage, and the activation of coagulation factors and platelets cause fibrin deposition in the microvasculature. These ultimately lead to the fragmentation of RBCs and the development of tumor-associated microvascular pathologic hemolytic anemia. The lack of responsiveness of bone marrow erythrocytes to erythropoietin (EPO) in the presence of cytokines, such as tissue necrosis factor α (TNF-α) and interferon γ (IFN-γ),also leads to the development of tumor-associated anemia (119). Anemia reduces the number of RBCs in the central column of the blood vessel, resulting in fewer platelets coming into contact with the endothelial surface surrounding the flowing blood column. As a result, impaired primary hemostasis and bleeding could occur (119). Furthermore, tumor-associated anemia can reduce the oxygen-carrying capacity of the blood, contributing to tumor hypoxia (120). Persistent tumor hypoxia can enhance malignant progression and increase tumor cell aggressiveness through clonal selection and genomic changes (121, 122).

As previously mentioned, cancer patients are more likely to have thromboembolism than healthy people. Thromboembolism is reported to be the leading cause of death in cancer patients who are receiving chemotherapy (123). In addition, the activation and hypercoagulability of platelets induced by cancer cells positively affect the development and spread of tumors. Patients with abnormally high hematocrit levels are at an increased risk for thrombotic disorders (124). There are several mechanisms involving erythrocyte-related platelet activation. Firstly, RBCs enhance platelet activation by releasing adenosine diphosphate (ADP). RBCs can directly modulate platelet reactivity through chemical signaling (125). RBCs can release ADP into the bloodstream under low pH, low oxygen pressure, hemolysis, RBC deformation, permanent RBC damage, shear stress, and as a response to mechanical deformation, causing platelet activation. Furthermore, activated platelets release granules containing ADP, which further promotes platelet activation (126–129). Ex vivo studies involving blood transfusion have also demonstrated that the release of ADP from RBCs promotes platelet activation and aggregation (130). Secondly, RBCs enhances platelet activation by reducing the bioavailability of Nitric Oxide (NO).Hemoglobin released from damaged RBCs enhances platelet activation and accelerates thrombus formation by decreasing NO bioavailability, preventing the suppressive effect of NO on platelet activation (131, 132). Damaged RBCs can also release arginase, which cleaves l-arginine and reduces NO release (131). Although the hemoglobin released during erythrocyte injury has been recognized as a transporter and a scavenger of NO, it has recently been shown that hemoglobin can also preserve the function of NO through the formation of S-nitrosothiols (133). S-nitrosothiols have anti-platelet activity similar to NO, so it can be concluded that injured erythrocytes may enhance platelet aggregation by scavenging NO and inhibit platelet activation by releasing functional NO equivalents (134). In addition to elucidating the role of hemoglobin(Hb) reactions with NO in platelet activation, Hb has also been shown to directly activate platelets (135), demonstrating that its platelet aggregation-promoting effect is due to the activation of platelets produced by ROSfrom the iron in Hb (136). And thirdly, RBCs also promote the marginalization of platelets. Under normal conditions, RBCs preferentially move down the center of blood vessels, promoting platelet margination, increasing platelet-endothelium interactions, and enhancing platelet adhesion and activation (137).In the disease state, abnormal erythrocytes and erythrocyte-derived microvesicles may also adhere to the extracellular matrix or endothelium, activate platelets and other cells, and enhance local thrombin production during thrombosis (117, 138).Furthermore, they can also interact with platelets, leading to the formation of erythrocyte-platelet aggregates (117, 139). RBCs also bind to platelets through ICAM-4 and fibrinogen to form erythrocyte-platelet aggregates that promote tumor thrombosis and stabilization (8, 140, 141). On the one hand, this interaction can further activate platelets and promote thrombosis. On the other hand, it can lead to the release of biochemical messengers and signal transduction ***via*** erythrocyte deformation, further activating platelets to promote their high expression of P-selectin,and enhancing platelet adhesion to tumor cells (142). In conclusion, platelet function can be amplified by the interaction of activated platelets with intacted red blood cells (142).

## 5 Platelet-monocyte interactions in tumors

Monocytes, the innate immune cells of the mononuclear phagocyte system, have emerged as important regulators of tumor development and progressions (143–145). When inflammation or tumors occur *in vivo*, monocytes and neutrophils are first attracted from the peripheral blood stream to the site of injury (146). Monocytes can then be recruited to pre-metastatic sites, interact with tumor cells, inhibit their adhesion, and promote natural killer (NK) cells to eliminate tumor cells by secreting chemokines including CCL3, CCL4, and CCL5 (147, 148).

### 5.1 Platelet-monocyte interactions and inflammation

Platelet-monocyte interactions have a tremendous impact on the inflammatory responses because such interactions are not only pro-inflammatory but also anti-inflammatory. In humans, three distinct monocyte populations were first classified through their morphology and differential expression of cluster of differentiation (CD) 14 and 16on their membrane surface (149). Classical monocytes express high levels of CD14 and no CD16 (CD14++CD16−), intermediate monocytes express high levels of both CD14 and CD16(CD14++CD16++),while non-classical monocytes express low levels of CD14 but high levels of CD16+ (CD14+CD16++) (149, 150). CD16+ monocytes are traditionally referred to as “pro-inflammatory” monocytes (151–153). Platelets are considered to be an immune regulator (3, 154) that can regulate immune system function by interacting with immune cells (2, 3, 60, 155, 156) or by releasing soluble mediators IL-1β, (TGF-β, PF4, MIP1α or RANTES). Normally, PLT preferentially binds monocytes (157–160), mainly by P-selectin (CD62P)-PSGL-1 (161). Platelets preferentially bind to intermediate and non-classical monocyte subtypes that express CD16. Furthermore, activated platelets are able to release large amounts of TGF-β, leading to upregulation of CD16 on monocytes and consequently inducing a transition from classical monocytes to intermediate and/or non-classical monocytes (162, 163).

Several factors, such as infection or injury, can contribute to the activation of both monocytes and platelets. Once activated, monocytes and platelets can interact directly through receptors expressed on their surfaces, then the soluble factors released from platelets can further modulate monocyte activity. This interaction can promote the recruitment and adhesion of monocytes or lead to platelet-monocyte complex formation (164). It has been proven that thrombin- activated platelets bind to circulating monocytes, thereby inducing the production of pro-inflammatory cytokines such as IL-8, TNFα, and IL-6,further promoting a pro-inflammatory phenotype (2, 165). *In vitro*, platelet release of TGF-β activates the p38 MAPK pathway, which induces *de novo* synthesis of cyclooxygenase-2 to promote inflammatory monocyte responses (166). Additionally, platelet-derivedHMGB1promotes the migration of monocytes to inflammatory tissues (101, 167). In addition, activated platelets release ADP from granules that bind to ADP receptors(or P2Y12receptors), prompting the rapid transfer of P-selectin to the platelet membrane and increasing the recruitment of neutrophils, monocytes, and lymphocytes to the tumor site (168). Gil-Bernabé found that platelet-tumor cell conjugates were also able to recruit monocytes to early metastatic niches by chemokines produced from platelets (e.g. RANTES) (169, 170). After this, recruited monocytes produce VEGF to improve the extravasation of tumor cells, thereby increasing vascular permeability (171) and promoting the formation of metastases (170, 172). Moreover,platelet factor 4 (PF4) drives monocyte migration by binding to the CCR1 receptor (173). Platelet-monocyte interactions also play a vital role in locally restricting inflammation. PF4 not only acts as an inflammatory mediator but also downregulates the chemotactic receptors CCR1, CCR5, and CCR2 on isolated human monocytes to interfere with monocyte migration (174). Contrary to their pro-inflammatory effect, platelets have also been shown to dampen pro-inflammatory responses by triggering IL-10 expression and downregulating TNF-α and IL-6 release by monocytes (2)and macrophages (175). These opposing effects of platelets on innate immune cells may be determined *via* the activation status of platelets (176, 177) or by the blood flow microenvironment (178). The underlying mechanisms for this need to be explored further in future studies.

### 5.2 Platelet-monocyte aggregation in tumors

Monocyte-platelet aggregates (MPA), are hallmarks of platelet and monocyte activation and play a potential important role in promoting inflammatory responses and thrombosis. It serves as a bridge between inflammation and thrombosis (151, 179, 180). PSGL-1-P-selectin interactions appear to be the initiating signal in monocyte-platelet aggregation and are further consolidated by enhancing its interactions with other receptorsand other downstream signals (2, 116, 181, 182). MPA recruits monocytes to tumor sites, regulates the inflammatory environment at primary and metastatic tumor sites, and influences tumorigenesis and cancer progression (4, 183, 184). In addition, MPA formation upregulates CD16 expression on monocytes, resulting in an enhanced pro-inflammatory effect (165). In contrast, additional data demonstrate that MPA enhances the expression of the anti-inflammatory cytokine IL-1,inhibiting the progression of inflammation (147, 178). However, future studies need to explore further the exact mechanisms underlying this opposing effect.Monocytes are the primary source of tissue factors in the blood and are a key element of the extrinsic coagulation cascade reaction. Monocytes actively bind to platelets, forming MPAs that are highly susceptible to thrombosis (185). Activated platelets rapidly trigger the surface exposure of TF in monocytes through the interaction of P-selectin with monocyte PSGL1,rapidly contributing to intravascular coagulation and thrombosis, and promoting tumor development and metastasis (186). Monocytes account for 16% of platelet thrombus-bound leukocytes, almost four times the proportion of monocytes in the circulating blood; this demonstrates the importance of MPAs in thrombosis (185). Platelets also interact directly with monocytes, facilitating increased expression of PSGL-1, CD40,CD11b, and CCR2 on the surface of monocytes (4, 163), which in turn enhances the formation of MPAs and promotes the recruitment of more monocytes to the endothelium (4, 181, 182, 187). In summary, MPA is involved in the inflammatory response, maintains blood in a hypercoagulable state, and play an vital role in tumorigenesis, cancer progression, and metastasis. Platelets and their interactions with monocytes also affect various stages of tumor development. However, the exact mechanisms and effects of MPA on tumorigenesis and cancer progression remain unclear and need to be explored further.

## 6 Platelet-macrophage interactions in tumors

Macrophages are immune cells that play a key role in innate immunity and are also involved in tissue repair and remodeling (188). Depending on the prevalent stimuli in inflammatory microenvironments, human macrophages can be differentiated from circulating monocytes *in vitro* and polarized towards a pro- or anti-inflammatory phenotype (typically recognized as classically activated type 1 [M1] and alternatively activated type 2 [M2] macrophages, respectively).

### 6.1 Types of macrophages and their role in tumors

M1 macrophages are induced by IFN-γ, bacteria-derived lipopolysaccharides (LPS),and TNF-αand are known to release cytokines that can promote inflammation and tissue damage (189–191). M1 macrophages also produce high levels of IL-12, IL-1, IL-23, TNF-α, CXCL10, and nitric oxide synthase (iNOS), which play important roles in the inhibition and killing of tumor cells. M1 macrophages also present tumor-specific antigens, indirectly inhibiting tumor growth (192).

In contrast, M2 macrophages are induced by IL-13 and IL-4and are well-known to release anti-inflammatory cytokines that promote tissue remodeling and inhibit inflammation (193, 194). M2 macrophages release epidermal growth factor, chemokines (such as CCL24and CCL22), and cytokines (such as TNF-α and IL-6) that directly promote tumor growth and metastasis and promote tumor progression. Meanwhile, several potent immunosuppressive factors produced by M2 macrophages (such as IL-10 and prostaglandin E2 [PGE2]) suppress anti-tumor immune responses and promote angiogenesis and stromal remodeling in the tumor microenvironment, ultimately promoting tumor development and metastasis (195). Tumor-associated macrophages mostly exhibit the M2 phenotype, and their significant contribution to tumor progression and metastasis has been noted (196). Platelets also play a key role in promoting macrophage polarization and consequently play a significant role in tumor progression.

### 6.2 Activated platelets induce monocyte differentiation into anti-inflammatory M2 macrophages

It has been found that monocyte incubation with activated platelets results in a biased differentiation into M2-type macrophages (197). Activated platelets produce TGF-β, which can convert M1 macrophages into M2 macrophages. Activated M2 macrophages produce small amounts of IL-12 and large amounts of IL-10, which suppress the inflammatory state of the body, attenuates the antineoplastic immune response, promote tumor angiogenesis, and accelerate tumorigenesis, development, and metastasis (198–205). ActivatedM2 macrophages also affect the T-cell inactivation, which severely reduces the body’s ability to resist cancer development and progression (205). In addition, platelet activation synthesizes lipid mediators such as thromboxane A2 (TXA2) (65, 206, 207) or PGE (208–210). PGE2directly induces IL-10 release from macrophages, decreases IL-6 and TNF-α release, controls tumor-associated inflammation, and decreases the body’s anti-tumor immune response (147, 176). In addition, activated platelets release PF4, which increases the levels of macrophage nuclear factor-kappa B (NF-κB), promotes MMP-9 gene transcription, and facilitates tumor vascular remodeling (210, 211). The release of vascular endothelial growth factor (VEGF) during PLT activation can also promote the recruitment of circulating macrophages to tumor sites (212–214).

### 6.3 Activated platelets also induce monocyte differentiation into pro-inflammatory M1 macrophages

Controversially, some studies have illustrated that platelets are able to modulate not only the pro-inflammatory but also the anti-inflammatory response of macrophages. Angiogenic factors and chemokines (e.g., CXCL4) may induce or modulate the pro-inflammatory response of macrophages during platelet activation (215). Increased levels of the pro-inflammatory cytokines IL-6,TNF-α, and IL-23, were observed after co-incubation of LPS- activated human monocyte-derived macrophages with activated autologous platelets, suggesting that the presence of activated platelets at the sites of inflammation may intensify pro-inflammatory macrophage activation (164). Other studies have shown that the presence of platelets skewed monocytes towards the M1phenotype in a cell-contact-dependent manner *via* the GPIb-CD11b axis in mice (196). However, given the differences between mouse and human macrophages, the biological relevance of platelet-macrophage interactions in human innate immunity remains uncertain.

## 7 Platelet- dendritic cell interactions in tumors

Dendritic cells (DCs), also known as “professional” antigen-presenting cells (APCs), can process and cross-present antigens to induce effective antigen-specific T-cell responses and are principal modulators of adaptive immunity (216). The role of dendritic cells in tumors has been well-investigated, and the effects of the interactions between platelets and dendritic cells on tumors have attracted significant attention.

### 7.1 Platelets promote DC maturation and activation

Platelets not only enhance the activity of macrophages and neutrophils but also affect the maturation and activation of DCs (217). Activated platelet-derived soluble CD40L (sCD40L or CD154) stimulates DC maturation and enhances antigen presentation capability. Platelets contribute to adaptive immunity by interacting with and activating DCs *via* the CD40-CD40L axis. The platelet-mediated activation of DCs results in antigen presentation to T-cells (3, 218, 219). Platelet-DC interactions also promote the over expression of co-stimulatory molecules on DCs and increase the production of cytokines such as TNF-α, IL-12, and IL-6.These cytokines promote the maturation of DCs to target T lymphocyte antigens that help activate type I helper T-cells (TH1 cells) and CD8^+^ T-cells to kill tumor cells (220). In addition, platelet-DC interactions may be regulated through the P-selectin/PSGL1 axis, and then platelets firmly adhere to CD11b/CD18 (MAC-1) on DCs *via* their junctional adhesion molecule C (JAM-C), resulting in DC activation and platelet phagocytosis (220).

### 7.2 Platelets inhibit DC maturation and activation

In contrast, some platelet-derived factors have the opposite effect, such as VEGF, serotonin/5-HT, PF4, TGF-β, and PGE2. Platelet-derived VEGF can inhibit antigen presentation in mature dendritic cells, thereby suppressing their immune surveillance function (221). In addition, 5-HT decreases the expression of DC co-stimulatory molecules and increases IL-10 levels, thereby reducing the ability of DCs to stimulate T-cells, suppress cellular immunity, and promote tumor cell evasion of immune cell killing (222). PF4 increases the responsiveness of DCs to TLR ligands (223) and reduced ability of dendritic cells to present antigens (224). TGF-β1is also a factor secreted by platelets that inhibits the activation and maturation of DCs *in vitro*. TGF-β1 inhibits the upregulation of key T-cell co-stimulatory molecules on the DC surface and reduces their antigen-presenting capacity (220). Moreover, platelets and platelet-derived soluble mediators inhibit the pro-inflammatory properties of DCs and even induce an anti-inflammatory phenotype, decreasing the T-cell priming capacity of DCs. This allows tumor cells to evade the killing effect of T-cells and promote tumor progression (225, 226).

Platelets exhibit complex interactions with DCs. These complex and sometimes contradictory interactions appear to directly affect tumor development. However, while platelets have been well-investigated in the context of thrombosis, hemostasis, and vascular inflammation, the potential mechanism and impactions of the interaction between platelets and dendritic cells in tumor development and progression are still unknown. Therefore, further studies are necessary to clarify the exact mechanism underlying platelet-DC interactions and their impact on tumor development.

## 8 Platelet-T-cell and platelet-B-cell interactions in tumors

T-cells mainly include CD8^+^ and CD4^+^cells. CD8^+^T-cells are also recognized to be cytotoxic. They recognize antigens presented in major histocompatibility complex class I molecules and cause tumor cell death through cytokine secretion. CD4+ cells are mainly responsible for regulating the immune response (60). They are further divided into various Th types, with Th1, Th2,and Th17 cells acting as immune effectors and T regulatory cells as immune suppressors.

### 8.1 Platelet-CD4^+^T-cell interactions in tumors

The communication between platelets and CD4^+^T-cells is similar to that between pre- and post-synaptic neurons. Platelets store large amounts of mediators in their vesicles that can be released rapidly, similar to the release of neurotransmitters from pre-synaptic neurons (3, 227, 228). Moreover, similar to post-synaptic neurons, CD4^+^T-cells also have multiple neurotransmitter receptors (e.g., dopamine and serotonin receptors) that provide a direct pathway through which platelets can immediately communicate (229–232). In addition to platelet-derived neurotransmitters, platelets release multiple soluble factors, integrins, and adhesion molecules during activation, affectingCD4^+^T-cell function.

Studies have shown that serotonin(5HT), CCL5,and platelet-activating factor(PAF) play important roles in activatingCD4^+^T-cells. First,CXCL4 found in platelets recruits and activates T-cells, and T cells can also activate platelets through CD40L interaction with the CD40 on platelets, leading to the release of plateletsCCL5 and further recruitment of T cells to prevent tumor progression (233, 234). Platelet-derived factors such asCCL5 and CXCL4 can also stimulate the proliferation and differentiation of enhance Th1 and Th17cells (235). In addition, platelet-derived 5HTcan stimulate CD4^+^T-cell proliferation and produce IFN-γ (232), promoting the activation and proliferation of naïve T-cells, enhancing cellular immunity, and increasing tumor cell cytotoxicity (236, 237). Furthermore, epinephrine has been shown to stimulate both Th17 and Th2 cells and produce CXCL1, which has been demonstrated to stimulate Th17 cells and may substitute or enhance the effect of other platelet-derived factors (238).

Research has confirmed that platelet-derived TGF-β inhibits the differentiation of immature T-cells to Th1 cells and promotes their differentiation into regulatory T lymphocyte (Treg) subpopulations (239). Tregs express glycoprotein A repetitions predominant (GARP), which binds and activates latent TGF-β (240–243) involved in enhancing the suppressive ability of Tregs and maintaining Treg-mediated peripheral tolerance (241, 244, 245). Studies have shown that the GARP-TGF-β complex, together with platelet-secreted lactate, inhibits T-cell responses against melanoma and colorectal cancer, thus exerting suppressive effects on anti-tumor immunity (239, 246). Platelet-derived microvesicles have been shown to inhibit IL-17 production by Treg cells through P-selectin. Moreover, IL-17 is considered to promote neovascularization, tumor cell proliferation, and immunosuppression (247). Therefore, platelets modulate the immunosurveillance processes during cancer development.

Besides stimulating CD4^+^T-cells *via* soluble factors, platelets also directly contact T-cells, thereby regulating the T-cell immune response. Studies have shown that when platelets have a reduced ability to produce pro-inflammatory factors and stimulate CD4^+^T-cells, their ability to form aggregates with CD4^+^T-cells is significantly increased. Platelets bind to CD4^+^T-cells through multiple receptors, including P-selectin (CD62P) and integrins (αIIb and βIII), forming platelet-CD4^+^T-cell aggregates that interfere with the interactions between CD4^+^T-cells and APCs (232, 248).

### 8.2 Platelet-CD8^+^T-cell interactions in tumor

The influence of interactions between platelets and CD8T cells on tumors is twofold. On the one hand, some studies have observed that metastatic tumors have lower numbers of CD8 T cells and higher frequencies of platelets than non-metastatic tumors, indicating that the interplay between platelets and CD8 T cells influences metastasis, and also confirmed the important role of platelet-MDSC-CD8 T-cell axis in it. Platelets secrete CXCL4 to induce Myeloid Derived Suppressor Cells(MDSC) production, and MDSCs negatively inhibits the function of CD8 T cells in return (249).. This facilitates the escape of tumor cells from the primary tumor site and into circulation resulting the establishment of metastasis. On the other hand, RANTES upregulates the cytotoxic function of CD8 + T-helper cells as well as cytokine production (250). Besides APCs attributed to their receptors and high expression of MHC-I on their surface (251), platelets are able to present antigens to naïve T-cells, effectively stimulate CD8+T-cells and promote them to exert cellular immunity and kill tumor cells (232, 252). Additionally, it has been demonstrated that in patients with lung cancer, platelets can attach to T-cells (more platelets attach to CD8^+^ cells than to CD4^+^ cells, and these platelets express P-selectin) and form platelet-T-cell aggregates to promote thrombosis, increasing the risk of death in these patients (253).

### 8.3 Platelet-B-cellinteractions in tumors

Platelets activate B-cells by releasing soluble CD40 and CD40L;increasing the production of IgG1, IgG2, and IgG3; inducing B-cell proliferation, differentiation, and allotype switching; memory B-cell production, and enhancing humoral immunity (254). In addition, platelets contain kappa-light chain enhancers of members of the activated B-cell NF-κB family of proteins (255) that influence inflammation and thrombosis (256). Moreover, platelet-lymphocyte interactions can promote lymphocyte recruitment to sites of inflammation infection or tumors and further mediate lymphocyte killing of tumors (257).

## 9 Platelet-natural killer cell interactions in tumors

**Figure 2:**
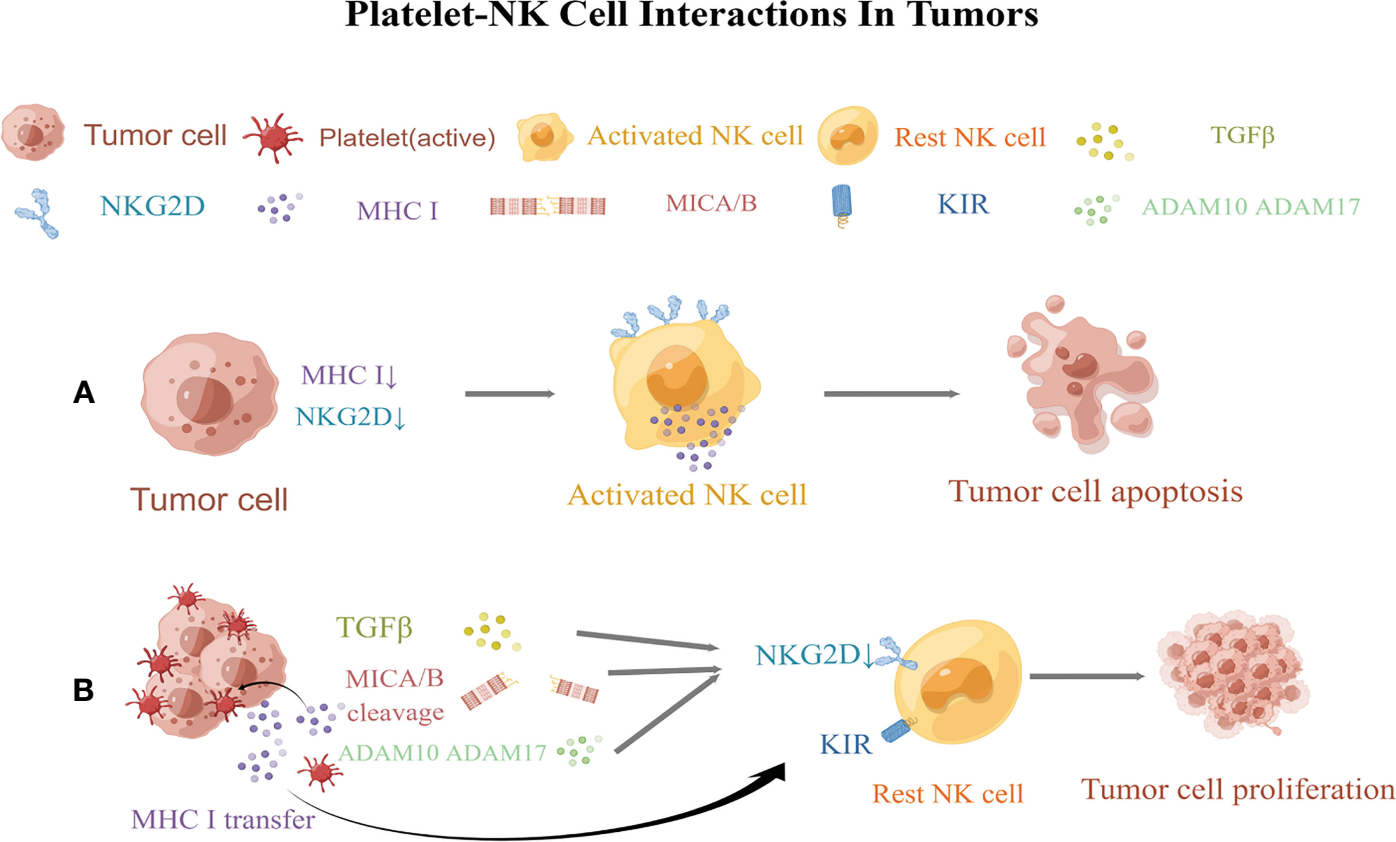
Regulation of natural killer (NK) cellsl responsiveness by platelets. **(A)** Normal NK cells kill tumor cells *via* the “missing self” and “induced self.” **(B)** Platelets obstruct NK cells, allowing tumor cells to proliferate by evading immune surveillance. Activated platelets then wrap around tumor cells, promote the transfer of MHC class I molecules to the surface of cancer cells, and decreases the “missing self” recognition of abnormal cells by NK cells. Platelets then reduce the expression of MICA and MICB on tumor cells and impair the “induced self” recognition of NK cells. Moreover, platelets also express the enzymes ADAM10 and ADAM17, which shed NKG2D on tumor cells. In turn, platelets release TGF-β to downregulate NKG2D and induce the expression of killer cell immunoglobulin-like receptors (KIRs) on NK cells. MICA and MICB, MHC class I chain-related protein **(A,B)** ADAM10andADAM17, a disintegrin and metalloproteinase domain-containing protein10 and 17; NKG2D,NK group 2D; KIRs, killer cell immunoglobulin-like receptors. This figure is drawn by Figdraw (https://figdraw.com/). The "↓" arrows refer to the decreased expression of MHCI and NKG2D.

NK cells play a central role in tumor immunosurveillance (258). NK cells are activated through the following mechanisms. First, the activation of NK cells is triggered by the recognition of the”missing self” of abnormal cells, where the “self” mainly refers to MHC class I molecules. “Missing self” means that NK cells preferentially destroy target cells with low or no expression of MHC class I molecules (259). Another mechanism is mediated by the “induced self”recognition of abnormal cells. “Induced self” refers to the expression of ligands used to activate NK receptors, especially NK group 2D (NKG2D), which enhances the cytotoxicity of NK cells (260). Platelets can inhibit NK cell recognition of abnormal cells by highly expressing MHC class I molecules and down regulating the expression and release of NKG2DL(NKG2D ligand) (6) (**Figure 2**).

### 9.1 Platelets weaken the “missing self” of NK cells

In the later stages of tumor development, malignant cells often metastasize *via* the bloodstream (261). However, owing to the abundance of NK cells in the blood (262), it is a hostile environment for tumor cell growth and metastasis. To resist this harsh environment, tumor cells release large amounts of cytotoxic factors to inhibit the killing effect of NK cells. More importantly, when tumor cells enter the circulation, platelets may provide an alternative mechanism to evade NK cell surveillance and increase the metastatic potential of tumor cells (57). The quantity and quality of platelets are directly proportional to the number of tumor cells that metastasize (263, 264). The depletion of platelets after intravenous inoculation of fibrosarcoma, lymphoma, and melanoma cell lines has been shown to directly inhibit metastasis in the lungs of mice with normal immune function, and this effect was reversed after the depletion of NK cells. This further indicates that platelets help circulating tumor cells evade immune surveillance by NK cells (58).Palumbo and his colleagues showed that tumor cells escape NK cell-mediated surveillance through fibrin deposition, thereby strengthening platelet aggregation on the surface of tumor cells (57). The ability of tumor cells to trigger platelet aggregation is related to the enhanced metastatic potential (58). Tumor cells can bind to platelet P-selectin *via* tumor cell-induced platelet aggregation, forming aggregates to protect themselves from circulation and to “hide” in the presence of NK cells (31). As reported previously, tumor cells were rapidly encapsulated in the presence of platelets *in vitro*, and the formation of a “platelet envelope” leads to the transfer of MHC class I to the surface of tumor cells, resulting in high levels of platelet-derived normal class I MHC expression on tumor cells. This “pseudoexpression” weakens the “missing self” recognition of abnormal cells by NK cells and reduces NK cell cytotoxicity (6, 56).

### 9.2 Platelets weaken the “induced self” of NK cells

In addition, the platelet coating on tumor cells reduces the surface expression of NKG2D ligands, including MHC class I chain–related proteins A and B(MICA and MICB) on tumor cells, impairing the “induced self” recognition of NK cells (265). It is widely known that tumor cells divide their own NKG2DL by expressing “a disintegrin and metalloproteinase domain-containing protein10 and 17” (ADAM10 and ADAM17) (266–268). Platelets can express both ADAM10 and ADAM17that can induce the shedding of NKG2D from tumor cells, thereby suppressing NK cell anti-tumor immune function (269–275). Additional studies have shown that the platelet-mediated release of TGF-β mediates NKG2DL downregulation and presents MHC class I molecules to NK cells *via* killer cell immunoglobulin-like receptor (KIR),which may result in the loss of cytotoxicity and IFN-γ-producing capacity of human NK cells (276). Platelets also express NKG2DL, specifically UL2-binding protein (ULBP2), which may be released in a soluble form. The bioactivity of soluble ULBP2 is unclear, however, its shedding might inhibit the NKG2D-inducedrecognition of platelet-cancer aggregates (277).

## 10 Platelet-endothelial cellinteractions in tumors

Endothelial cells (ECs) are situated on the inner surface of blood vessels, therefore in immediate contact with blood components and cells. ECs are also important for controlling blood fluidity; leukocyte activation, adhesion and migration; and platelet adhesion and aggregation (278).

### 10.1 Platelet-endothelial cell interactions promote thrombosis

Healthy ECs release prostacyclin (PGI2) and NO that counteract platelet activation, prevent clotting and thrombosis, and maintain unobstructed blood flow and tissue perfusion (279, 280). After endothelial injury, platelets were gathered in the damaged area of endothelium, quickly adhered and activated, resulting in the release of α-granules and the activation of integrin αIIbβ3 (281, 282). The von Willebrand factor (VWF) is essential for platelet adhesion. It is a multimeric glycoprotein synthesized by ECs and stored in Weibel-Palade bodies. VWF is located in α-granules of platelets and is also synthesized by megakaryocytes (283). Injured ECs release VWF, which acts as a bridge between platelets and subendothelial collagen *via* glycoprotein (GP)-Ibαon the platelet surface; it is also involved in platelet aggregation and thrombosis (283–285). Another function of the VWF is to act as a carrier and stabilizer of plasma FVII in circulation and to protect it from degradation by activated protein C by forming the VWF-FVII complex (286–288). VWF also interacts with platelet GPIbα, enhancing integrin β2-mediated interactions between platelets and neutrophils (278, 289) and coordinating the activation of neutrophils, platelets, and coagulation cascades that promote tumorigenesis and progression. In addition, collagen interacts with platelets *via* integrin α2β1 and GPVI to induce intracellular signaling and enhance platelet adhesion. The platelet fibrinogen receptor integrin αIIbβ3 strengthens platelet adhesion and promotes thrombosis (290). Moreover, platelets and ECs synthesize PAF, which promotes platelet adhesion to ECs as well as their activation and aggregation (291).

### 10.2 Platelet-endothelial cell interactions promote tumor angiogenesis

Growing tumors require large amounts of nutrients and oxygen, but a lack of contact with their internal vascular network often leads to hypoxia. Thus, angiogenesis is a crucial stage of cancer progression. The α-particles of platelets are rich in vasoactive substances and chemokines, such as serotonin,TXA2, PAF, platelet-derived growth factor-BB (PDGF-BB) and VEGF (292, 293). When platelets are activated, it can not only release PDGF-BB to promotes the upregulation of VEGFgeneexpression in vascular ECs, but also directly releases VEGF promoting the proliferation, differentiation, and migration of vascular ECs, enhances the permeability of vascular ECs, promotes neovascularization, provides oxygen and nutrients to ischemic and hypoxic tissues, and causes the rapid growth of tumor cells (7, 294, 295). The role of VEGF in neoplastic diseases also leads to enhanced TF expression and increased VWF release in ECs, thereby promoting thrombus formation and development at tumor sites (296). Platelets also indirectly promote leukocyte migration by activating ECs and inducing the secretion of Weibel-Palade vesicles from EC, leading to CD62P expression and IL-8 release. In turn, the release of these molecules leads to neutrophil rolling, adhesion, and extravasation and promotes the tumor inflammatory microenvironment and tumor development (289).

## 11 Summary

**Figure 3:**
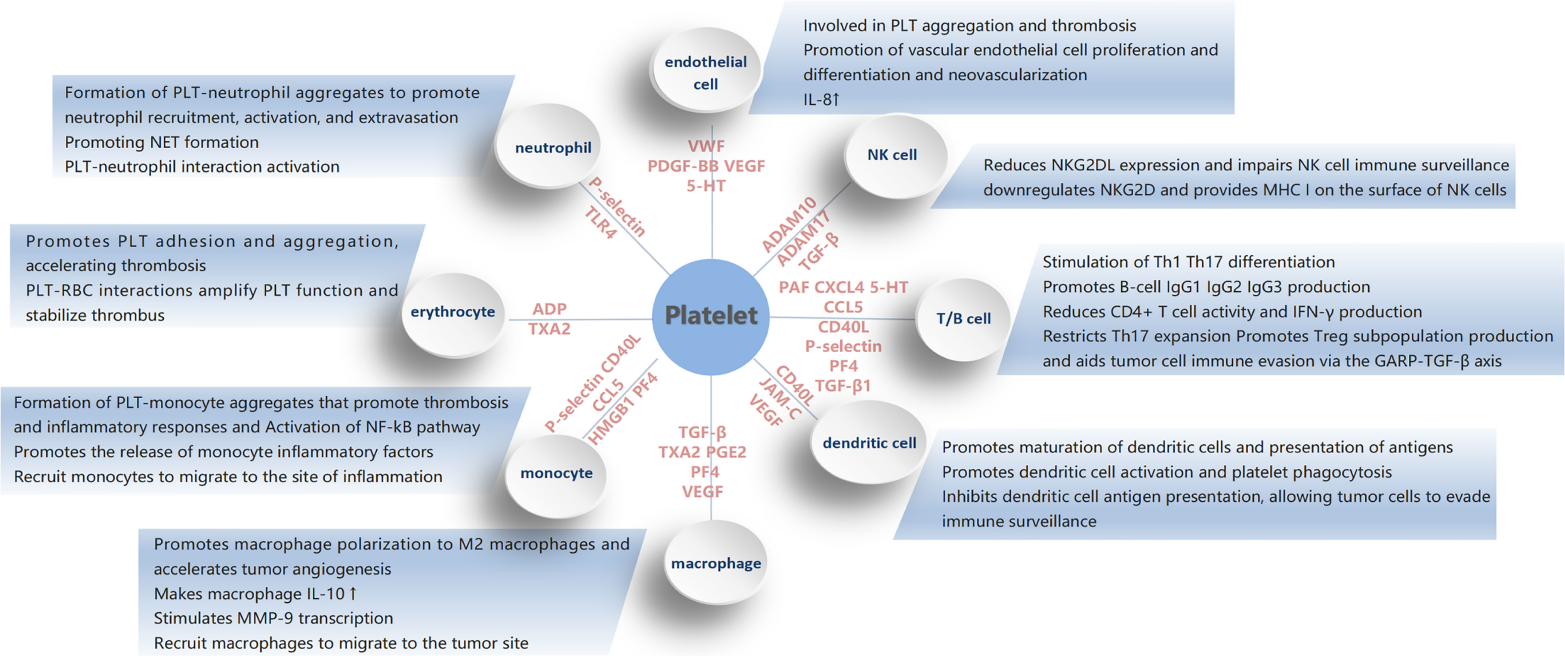
The figure shows the mechanism of platelet action with various immune cells.

Platelets play an important role in hemostasis and thrombosis, and they also play a key role in tumorigenesis and tumor development by interacting with all cells in the tumor microenvironment (**Figure 3**). It affects various biological processes in the disease state. Studies have shown that platelets are involved in the immune response, regulate the tumor microenvironment, and are closely associated with the pathophysiology of thrombosis, inflammation, and tumor progression. The mechanisms of platelets interacting with cells in the tumor microenvironment are complex and appear to have a dual behavior, pro- cancer and anti-cancer. Various platelet-cell interactions, in turn, lead to continuous platelet activation, further promote tumor development.

In conclusion, better understanding of the interaction between platelets and cells in the tumor microenvironment is helpful to understanding the mechanisms of the dual role of platelets in promoting and suppressing cancer, and future studies concerning these questions will provide a new direction for cancer prevention and therapy.

## Author contributions

ZZ and YY performed the scientific literature search, collected and analyzed data, designed the review structure and wrote the manuscript. ZM and LQ prepared the tables and figures. All authors contributed to the article and approved the submitted version.
